# The Epidemiological Characteristics of the Korean Bat Paramyxovirus between 2016 and 2019

**DOI:** 10.3390/microorganisms8060844

**Published:** 2020-06-04

**Authors:** Seong Sik Jang, Ji Yeong Noh, Van Thi Lo, Yong Gun Choi, Sun-Woo Yoon, Dae Gwin Jeong, Hye Kwon Kim

**Affiliations:** 1 Department of Microbiology, College of Natural Sciences, Chungbuk National University, Cheongju 28644, Korea; jss4724@naver.com (S.S.J.); wldud1540@chungbuk.ac.kr (J.Y.N.); 2 Infectious Disease Research Center, Korea Research Institute of Bioscience and Biotechnology, Daejeon 34141, Korea; van@kribb.re.kr (V.T.L.); syoon@kribb.re.kr (S.-W.Y.); dgjeong@kribb.re.kr (D.G.J.); 3 Bio-Analytical Science Division, University of Science and Technology (UST), Daejeon 34113, Korea; 4 The Korean Institute of Biospeleology, Daejeon 34225, Korea; kcavere@hanmail.net

**Keywords:** bat, paramyxovirus, *Shaanvirus*, Korea

## Abstract

Bats are considered reservoirs of severe emerging human pathogens. Notably, bats host major mammalian paramyxoviruses from the family Paramyxoviridae, order Mononegavirales. In this study, paramyxoviruses were investigated by reverse transcription semi-nested polymerase chain reaction (RT-semi-nested PCR) and reverse transcription polymerase chain reaction (RT-PCR), based on the RT-semi-nested PCR using the consensus paramyxovirus primers targeting the RNA dependent-RNA-polymerase (RdRp) region. In addition, RT-PCR was performed using newly designed primers targeting regions of the fusion protein (F) and hemagglutinin-neuraminidase (HN). The dominant bat species in the collection site of paramyxoviruses were *Miniopterus schreibersii*, *Myotis macrodactylus, Myotis petax*, and *Rhinolophus ferrumequinum*. Paramyxoviruses were detected in four samples in 2016 and six in 2019. Meanwhile, in samples collected in 2017 and 2018, no paramyxoviruses were detected. Phylogenetic analysis based on the partial nucleotide sequences of RdRp, F, and HN proteins suggested that the viruses belonged to the proposed genus *Shaanvirus*. In conclusion, this study revealed that bat paramyxoviruses in Korea belonged to a single genus and circulated sporadically in several provinces, including Chungbuk, Gangwon, Jeju, and Jeonnam.

## 1. Introduction

Severe acute respiratory syndrome coronavirus (SARS-CoV), the Middle East respiratory syndrome coronavirus (MERS-CoV), and the Ebola virus are all thought to be bat-borne viruses. In addition, the recent acute respiratory disease caused by SARS-CoV-2 is also suspected as a bat-borne virus [1]. These bat-borne viruses are primarily transmitted to humans via intermediate host animals. For SARS-CoV, this intermediate host is believed to be palm civets, while camels play that role for MERS-CoV [2,3]. The zoonotic host of the Nipah virus belonging to the paramyxovirus is the *Pteropus* bat and the pig is likely an amplifying host [4]. However, Nipah virus transmission from bat to human via date palm sap contaminated with urine from infected bats was identified [5,6]. Further, epidemiologic investigation of Nipah virus-associated outbreaks implicated human-to-human transmission [7]. Hence, Nipah virus was included in the WHO R&D Blueprint list to prevent public health emergencies. In addition, serological evidence of possible human infection by the Tioman virus belonging to the paramyxovirus enhances the role of bats in the epidemiologic role of pathogens [8].

The paramyxovirus family consists of several viruses having very broad host ranges [9]. Paramyxoviruses have been isolated from different vertebrates, including fish and mammals [10]. Among various vertebrates, bats host major mammalian paramyxoviruses from the subfamily Orthoparamyxovirinae, family Paramyxoviridae, and order Mononegavirales [11,12]. The Hendra and Nipah viruses are emerging bat-borne infectious agents that are highly pathogenic paramyxoviruses, which have caused outbreaks of respiratory and neurological diseases in humans and domesticated mammals [13,14]. 

Novel paramyxoviruses are increasingly being reported around the world, with the identification of three new genera (*Jeilongvirus*, *Narmovirus*, *Salemvirus*) being created from the subfamily Orthoparamyxovirinae by the International Committee on Taxonomy of Viruses (ICTV) [11]. Recent bat-associated paramyxoviruses were classified as a separate phylogenetic clade proposed as *shaanvirus* [15]. In 2018, the isolation and genetic characterization of a bat paramyxovirus in Korea was reported. Phylogenetic analysis showed that the virus belonged to the proposed *shaanvirus* clade [16]. However, there have been very few studies conducted on bat paramyxoviruses in Korea, except for the 2018 study. 

Therefore, we investigated paramyxoviruses in Korean bat species using 473 fecal samples collected from 2016 to 2019 at 85 sites in natural bat habitats.

## 2. Materials and Methods

### 2.1. Samples

To avoid animal disturbance, the study was focused on the analysis of guano collected beneath bat roosting sites/colonies in Korea. This sampling design did not require ethical approval for the study. From 2016 to 2019, 473 fecal samples were collected at 85 sites in natural bat habitats (Table S1). We sampled fecal pellets directly on the cave floor with sterile swabs; fresh fecal samples were preferred. The fecal samples were immediately placed into Universal Transport Medium (Noble Biosciences™, Hwaseong, Korea). The samples were transported to the laboratory and ultimately stored at –80 °C until the analysis.

### 2.2. Reverse Transcription Semi-Nested PCR (RT-Semi-Nested PCR) and Reverse Transcription PCR (RT-PCR) Screening

RNA was extracted from the bat raw samples using Trizol LS (Invitrogen, Carlsbad, CA, USA). cDNA was synthesized using an M-MLV reverse transcriptase kit (Promega, Madison, WI, USA) with a mixture of random hexamer. RT-semi-nested PCR was performed using a consensus paramyxovirus primer targeting the RNA-dependent-RNA-polymerase (RdRp) region [15]. In addition, RT-PCR was performed with newly designed primers targeting regions of the fusion protein (F) and hemagglutinin-neuraminidase (HN) (Table 1). In this study, we used bat paramyxovirus B16-40 (accession no. MG230624.1) belonging to the proposed genus *shaanvirus* as a positive control and nuclease free water as a negative control.

For the first amplification in the semi-nested assay, the optimized PCR mixture contained 10 μL Accupower^®^ PCR MasteMix (Bioneer, Daejeon, Korea), 7 μL nuclease free water, 1 μL each of 10 pmol forward and reverse primers, and 1 μL cDNA. The mixture was first heated to 95 °C for 5 min, followed by 35 cycles at 95 °C for 20 s, 50 °C for 20 s, 72 °C for 30 s, and a final extension at 72 °C for 5 min. For the second amplification, we used 10 μL Accupower^®^ PCR MasterMix, 7 μL nuclease free water, 1 μL each of 10 pmol forward and reverse primers, and 1 μL aliquot from the first reaction and the conditions were the same. 

RT-PCR was performed to detect F and HN regions of paramyxoviruses. Each reaction mixture contained 10 μL Accupower^®^ PCR MasterMix, 7 μL nuclease free water, 1 μL each of 10 pmol forward and reverse primers, and 1 μL cDNA. For the detection of the F region, the mixture was first heated to 95 °C for 5 min, followed by 40 cycles at 95 °C for 20 s, 49 °C for 30 s, 72 °C for 40 s, and a final extension at 72 °C for 5 min. For the detection of HN region, the mixture was first heated to 95 °C for 5 min, followed by 40 cycles at 95 °C for 20 s, 50 °C for 30 s, 72 °C for 40 s, and a final extension at 72 °C for 5 min. The PCR products were revealed by electrophoresis on 1% agarose gel and the gels were purified using ExpinTM Combo GP mini kit (GeneAll, Seoul, Korea). The PCR products were submitted to the Cosmogenetech company in Daejeon, Korea, for sequencing. The nucleotide data obtained from Sanger sequencing of the PCR fragments were further analyzed with related sequences in BLASTn using BioEdit software version 7.2.5 [18].

### 2.3. Phylogenetic Analysis

The nucleotide sequences were aligned by the ClustalW module using BioEdit. Each sequence was trimmed to where both forward and reverse reads were a 100% match with the consensus sequence. The phylogenetic analyses were conducted with the maximum-likelihood method with 1000 replicates of bootstrap sampling and the Kimura 2-parameter model using MEGA version X [19].

### 2.4. Bat Species Identification

According to a recent report, there are 23 species of bats in Korea. Most Korean bats are thought to be insectivores, as no fruit bats have been found in Korea to date [20] due to the fecal samples collected from the cave which is the harbor for multiple bat species. DNA extraction was performed only for PCR positive individuals. DNA was extracted from the bat raw samples using QIAmp^®^ DNA Mini kit (QIAGEN, Hilden, NRW, Germany). PCR was performed using a specific primer targeting the cytochrome b gene. Forward (5′-TCATCMTGATGAAAYTTYGG-3′) and reverse (5′-ACTGGYTGDCCBCCRATTCA-3′) primers targeting a 946 bp region of the cyt b gene were used. The optimized PCR mixture contained 10 μL Accupower^®^ PCR MasteMix, 6 μL nuclease free water, 1 μL each of 10 pmol forward and reverse primers, and 2 μL DNA. The mixture was first heated to 95 °C for 5 min, followed by 40 cycles at 95 °C for 30 s, 47 °C for 30 s, 72 °C for 1 min, and a final extension at 72 °C for 10 min [21]. The amplicons from positive PCRs were sequenced using target-specific forward and reverse primers synthesized by Cosmogenetech Co. Ltd. (Daejeon, Korea). The nucleotide data obtained from Sanger sequencing of the PCR fragments were further analyzed with related sequences in BLASTn using BioEdit.

## 3. Results

### 3.1. PCR-Based Detection of Bat Paramyxoviruses in Bat Feces

In total, 473 samples were tested for the presence of paramyxovirus by nested RT-PCR detection of an RdRp gene fragment. Paramyxoviruses were detected in 2.1% (10/473) of samples obtained from the bat natural habitats in 85 sites.

**Figure 1:**
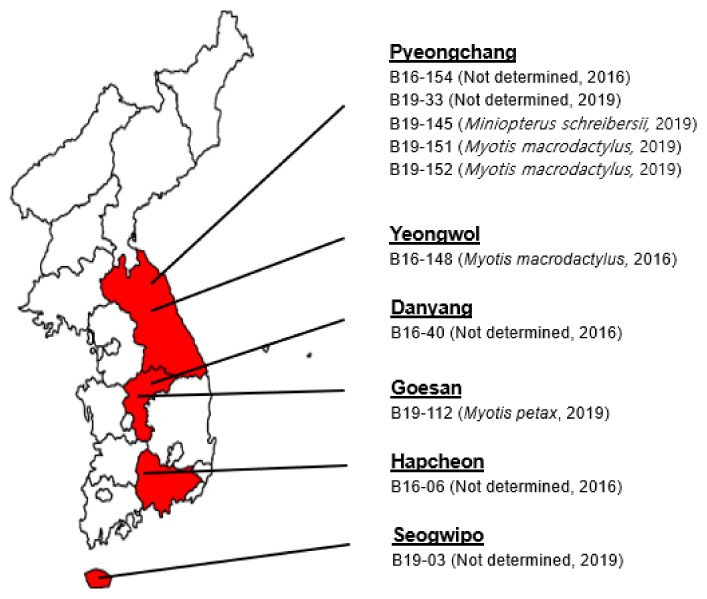
A map of sampling sites for positive samples. Samples were collected from around the country. Positive samples were detected in several provinces, including Gangwon, Chunbuk, Jeonnam and Jeju. In the Pyeongchang and Yeongwol Regions of Gangwon Province, bat paramyxovirus was detected in five and one samples, respectively. In the Danyang and Goesan Regions of Chungbuk Province, bat paramyxovirus was detected in one sample, respectively. In the Hapcheon Region of Jeonnam Province, bat paramyxovirus was detected in one sample. In the Seogwipo Region of Jeju Island, bat paramyxovirus was detected in one sample.

In 2016, 121 bat fecal samples were collected and four positive samples were identified based on RT-semi-nested PCR using consensus primers targeting the RdRp region. B16-6 and B16-40 were obtained at the BT cave in Hapcheon in March 2016 and G cave in Danyang in April 2016, respectively. The detection rate in March was 7.7% (1/13) and in April 20% (1/5). B16-148 was obtained at the OJ cave in Yeongwol in July 2016. B16-154 was obtained at the S cave in Pyeongchang in July 2016 (Table S1 and Figure 1). The detection rate in July was 6.7% (2/30) (Table 2 and Table S2). 

A total of 214 bat fecal samples were collected between 2017 and 2018 but no samples were positive for paramyxoviruses (Table 2).

In 2019, 138 bat fecal samples were collected and six positive samples were identified based on RT-semi-nested PCR using consensus primers targeting the RdRp region. B19-3 was obtained at the SAOL cave in Seogwipo in February 2019. B19-33 was obtained at the GCS cave in Pyeongchang in April 2019. B19-112 was obtained at the LK cave in Goesan in July 2019. B19-145 was obtained at the JA cave in Pyeongchang in August 2019. B19-151 and B19-152 were obtained at the S cave in Pyeongchang in August 2019 (Table S1 and Figure 1). Positive samples were detected in February, April, July, and August with each detection rate of 12.5% (1/8), 6.7% (1/15), 3.1% (1/32) and 12% (3/25) (Table 2 and Table S2).

In addition, RT-PCR was performed only for RT-semi-nested PCR positive individuals, based on the RT-PCR using the newly designed paramyxovirus primers targeting the F and HN region. Two samples, B19-33 and B19-152, and one sample, B19-151, were positive, respectively.

The GenBank accession numbers for the nucleotide sequence in this study are MT212726, MT230548, MT264743-MT264753, and MT277365.

### 3.2. PCR-Based Identification of Bat Species in Bat Feces

A total of 10 paramyxoviruses were detected in bat fecal samples collected from 2016 to 2019. To identify the bat species, PCR was performed using a consensus primer targeting the cyt b gene. Cyt b sequences of approximately 946 bp were obtained from B16-148, B19-112, B19-145, B19-151, and B19-152. The major bat species were identified as *Miniopterus schreibersii*, *Myotis macrodactylus*, *Rhinolophus ferrumequinum,* and *Myotis petax*. In this study, five bat paramyxovirus samples (B16-148, B19-112, B19-145, B19-151, and B19-152) were identified as bat species by PCR through special primer pairs but the others could not be identified because there were no remaining bat raw samples (Table S2).

### 3.3. Phylogenetic Analysis Based on the Genomic Nucleotide Sequence of the Paramyxovirus

**Figure 2:**
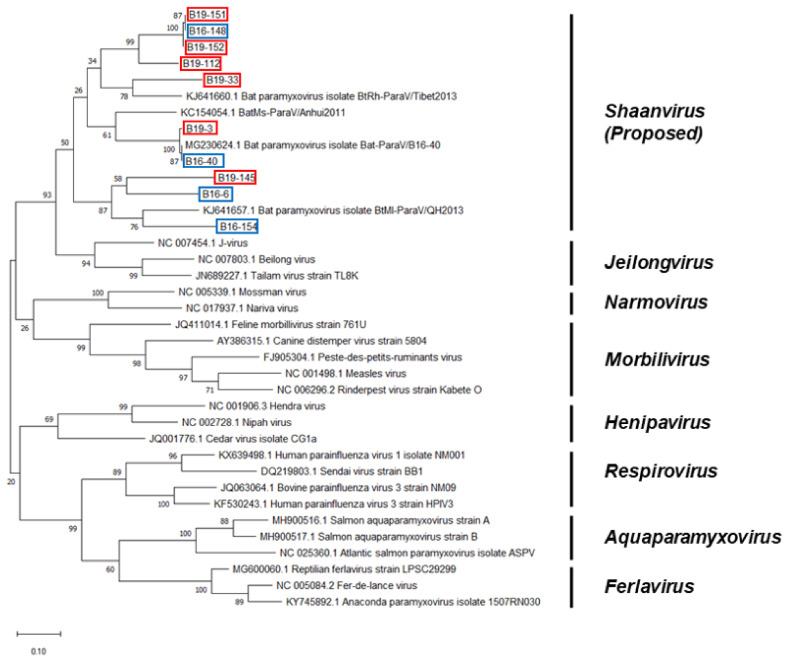
Phylogenetic analysis based on partial RNA-dependent-RNA-polymerase (RdRp) nucleotide sequences of bat paramyxoviruses detected in Korean bats and other reference viruses belonging to *Paramyxoviridae*. The phylogenetic tree was generated via the maximum-likelihood method with 1000 replicates of bootstrap sampling and Kimura 2-parameter model using MEGA X. Korean bat paramyxoviruses detected in 2016 are indicated by blue boxes. Korean bat paramyxoviruses detected in 2019 are indicated by red boxes.

Partial RdRp sequences were obtained from B16-6, B16-40, B16-148, B16-154, B19-3, B19-33, B19-112, B19-145, B19-151, and B19-152. The lengths of the sequences were 535, 467, 463, 465, 469, 487, 470, 457, 464 and 466 bp, respectively. The sequences of the partial RdRp region of the bat paramyxoviruses were analyzed with other paramyxovirus sequences from the GenBank database. The Korean bat paramyxoviruses belong to the proposed genus *Shaanvirus* and showed 68.99–100% nucleotide identity with the paramyxovirus isolated in Korea (Bat-ParaV/B16-40) and 69.75–77.69% nucleotide identity with the paramyxoviruses isolated in China (Bat-ParaV/Anhui2011 and Bat-ParaV/QH2013) (Figure 2). In the BT cave, one of nine fecal samples (B16-6) was found to be a bat paramyxovirus showing 71.02% nucleotide identity with Bat-ParaV/Anhui2011 (GenBank accession no. KC154054.1). In the G cave, one of 18 fecal samples (B16-40) was found to be a bat paramyxovirus showing 100% nucleotide identity with Bat-ParaV/B16-40 (GenBank accession no. MG230624.1). In the OJ cave, one of 12 fecal samples (B16-148) was found to be a bat paramyxovirus showing 74.46% nucleotide identity with Bat-ParaV/B16-40. In the S cave, three of 18 fecal samples (B16-154, B19-151, and B19-152) were found to be a bat paramyxovirus showing 76.51% nucleotide identity with Bat-ParaV/QH2013 (GenBank accession no. KJ641657.1) and 74.24%–74.89% nucleotide identity with Bat-ParaV/B16-40. In the SAOL cave, one of 25 fecal samples (B19-3) was found to be a bat paramyxovirus showing 98.72% nucleotide identity with Bat-ParaV/B16-40. In the GCS cave, one of 26 fecal samples (B19-33) was found to be a bat paramyxovirus showing 74.41% nucleotide identity with Bat-ParaV/B16-40. In the LK cave, one of six fecal samples (B19-112) was found to be a bat paramyxovirus showing 74.36% nucleotide identity with Bat-ParaV/Anhui2011. In the JA cave, one of 35 fecal samples (B19-145) was found to be a bat paramyxovirus showing 74.07% nucleotide identity with Bat-ParaV/QH2013.

**Figure 3:**
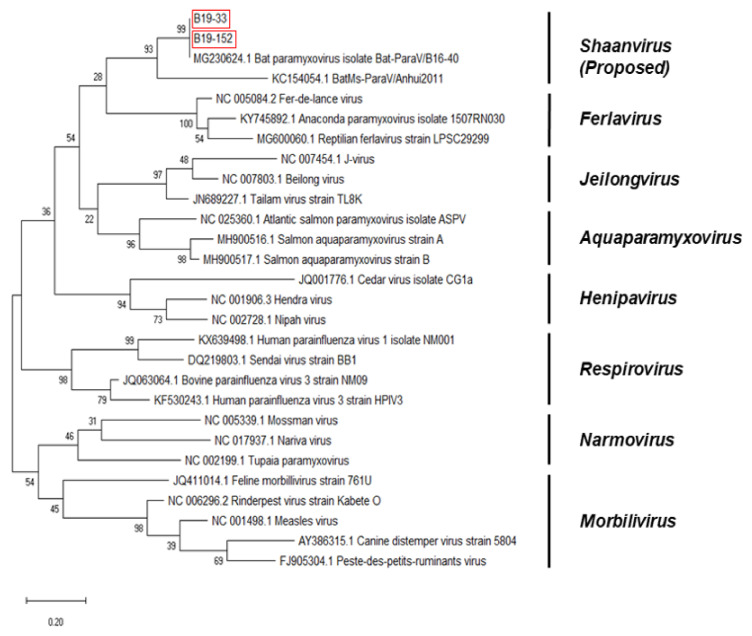
Phylogenetic analysis based on partial Fusion protein (F) nucleotide sequences of bat paramyxoviruses detected in Korean bats and other reference viruses belonging to *Paramyxoviridae*. The phylogenetic tree was generated via the maximum-likelihood method with 1000 replicates of bootstrap sampling and Kimura 2-parameter model using MEGA X. Korean bat paramyxoviruses are indicated by red boxes.

Partial F sequences were obtained from B19-33 and B19-152. The lengths of the sequences were 224 and 220 bp, respectively. The sequences of the partial F region of the bat paramyxoviruses were analyzed with other paramyxovirus sequences from the GenBank database. The Korean bat paramyxoviruses belonged to the proposed genus *Shaanvirus* and showed 93.75–100% nucleotide identity with the paramyxovirus isolated in Korea (Bat-ParaV/B16-40) and 93.75% nucleotide identity with the paramyxovirus isolated in China (Bat-ParaV/Anhui2011) (Figure 3). B19-33 and B19-152 were found to be a bat paramyxovirus showing 100% and 99.56% nucleotide identity with Bat-ParaV/B16-40, respectively.

**Figure 4:**
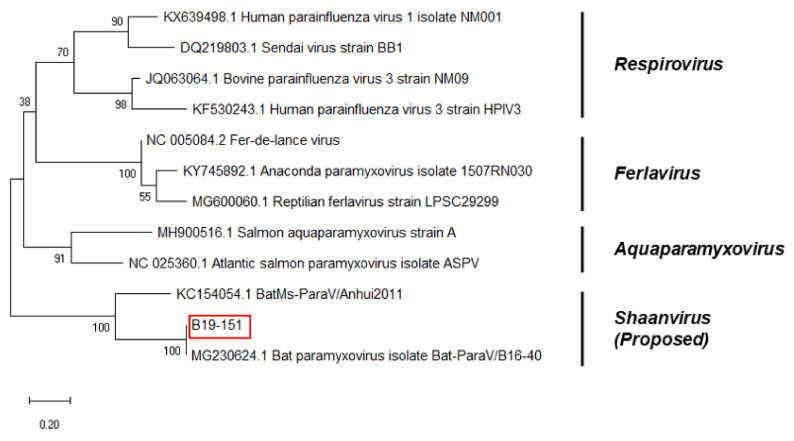
Phylogenetic analysis based on partial Hemagglutinin Neuraminidase (HN) nucleotide sequences of bat paramyxoviruses detected in Korean bats and other reference viruses belonging to *Paramyxoviridae*. The phylogenetic tree was generated via the maximum-likelihood method with 1000 replicates of bootstrap sampling and Kimura 2-parameter model using MEGA X. Korean bat paramyxoviruses are indicated by red boxes.

A partial HN sequence was obtained from B19-151. The length of the sequence was 245 bp. The sequence of the partial HN region of the bat paramyxovirus was analyzed with other paramyxovirus sequences from the GenBank database. The Korean bat paramyxovirus belonged to the proposed genus *Shaanvirus* and showed 100% nucleotide identity with the paramyxovirus isolated in Korea (Bat-ParaV/B16-40) and 92.86% nucleotide identity with the paramyxovirus isolated in China (Bat-ParaV/Anhui2011) (Figure 4).

Based on the partial RdRp, F, and HN nucleotide sequences, the Korean bat paramyxoviruses belonged to the proposed genus *shaanvirus*. These data indicated a single, closely related group of paramyxoviruses circulating in Korea.

## 4. Discussion

Bats are considered natural reservoirs for various zoonotic viruses [9]. Notably, bats host major mammalian paramyxoviruses [9,10,12]. In this study, we screened for bat paramyxoviruses in Korea and discovered 10 paramyxoviruses between 2016 and 2019. The National Institute of Biological Resources have reported 23 species of bat present in Korea [20]. We identified four bat species (*Miniopterus schreibersii*, *Myotis macrodactylus, Myotis petax*, and *Rhinolophus ferrumequinum*) based on PCR. We identified a bat species with a consensus primer that targets cyt b gene. Cyt b sequences of approximately 946 bp were obtained from B16-148, B19-112, B19-145, B19-151, and B19-152 [21].

Bat paramyxoviruses have been consistently detected in Korea. Four paramyxoviruses were detected from bat fecal samples in 2016. Paramyxoviruses were not detected in bat fecal samples in 2017 and 2018. Six paramyxoviruses were detected in bat fecal samples in 2019 (Table 2). Overall, 107 bat fecal samples were collected from natural bat habitats in 2017 and 2018, respectively. Some samples were collected at the same site where the paramyxovirus was detected but there were no positive samples in 2017 and 2018. It is expected that there is an active cycle of Korean bat paramyxoviruses. 

The phylogenetic analysis based on partial RdRp nucleotide sequences indicated that Korean bat paramyxoviruses belonged to the unclassified proposed genera *Shaanvirus* (Figure 2). The maximum likelihood tree showed that Korean bat paramyxoviruses were closely related to the bat paramyxovirus ParaV/Anhui2011, BtMI-ParaV/QH2013, and Bat-paraV/B16-40. The newly identified bat paramyxoviruses in China, ParaV/Anhui2011 and BtMI-ParaV/QH2013, were recently proposed to be part of the Genus *shaanvirus* [15]. In addition, isolation of Bat-paraV/B16-40 occurred in the Korean bat *Miniopterus schreibersii* in a previous study in 2018 [16]. Additionally, the phylogenetic analysis based on partial F and HN nucleotide sequences showed that Korean bat paramyxoviruses are closely related to the single clade (Figure 3 and Figure 4).

B16-154, B19-151, and B19-152 are bat paramyxoviruses detected in the same cave. It was interesting that there were differences in the partial RdRp nucleotide sequence of bat paramyxoviruses detected in 2016 and 2019. Although the bat species of B16-154 sample is unclear, these results suggest the possibility of genetic diversity in the same place.

Although our results have limitations that the partial nucleotide sequence used and some information of the associated bat species were not clearly available, this study described the existence of bat paramyxovirus in Korea. Furthermore, our results revealed that paramyxoviruses circulated in bat species in Korea. Based on our discovery of paramyxoviruses in bats, it is implied that there are many more paramyxoviruses circulating in bat species and various geographic areas.

Contact between humans and wildlife was identified as the origin of the recent epidemics [22,23]. In Korea, there is usually limited contact with bats because they roost in caves or deep inside forests. However, some caves where bats roost are located near human habitats. People frequently visit caves to avoid hot weather and store fermented foods. Close human-bat interactions may lead to human exposure to zoonotic pathogens [24]. In addition, the size of bat habitats is decreasing due to indiscriminate forest destruction. The resulting bat movement increases the chances of contact with humans as well as livestock. Zoonotic pathogens in bats have the potential to be transmitted to humans and livestock. We do not know whether the paramyxoviruses detected in this study are zoonotic pathogens. To determine this, follow-up studies involving virus isolation, characterization, and genomic analysis to identify their prevalence, epidemiology and zoonotic potential are required.

In conclusion, we have detected the presence of bat paramyxoviruses and revealed that paramyxoviruses circulated in bat species in Korea. Although the partial sequence was confirmed in this study, we confirmed the diversity of the detected nucleotide sequence of bat paramyxoviruses. In addition, the phylogenetic analysis based on the partial nucleotide sequences of RdRp, F, and HN proteins suggested that the viruses belonged to the proposed genus *Shaanvirus*. 

## Figures and Tables

**Table 1:** Information regarding the primers for RT-semi-nested PCR and RT-PCR.

Target	Primer	Sequence (5′-3′)	PCR Method	Amplicon Size (bp)	Reference
RdRp	PAR-F1	GAAGGITATTGTCAIAARNTNTGGAC	RT-semi-nested PCR	580	[17]
	PAR-F2	GTTGCTTCAATGGTTCARGGNGAYAA			
	PAR-R	GCTGAAGTTACIGGITCICCDATRTTNC			
F	F-F2	ACATCAGCCCAGATTACTGC	RT-PCR	320	This study
	F-R2	AGCTTGAATTGACAAGGTCT			
HN	HN-F2	CCTAATAAACTCAGCATCAAG	RT-PCR	350	This study
	HN-R2	GCTATCTGGTTGTGAGTGTA			

**Table 2:** Number of positive samples in each month from 2016 to 2019.

	Month	Jan	Feb	Mar	Apr	May	Jun	Jul	Aug	Sep	Oct	Nov	Dec	Total
Year														
**2016**		-	-	1/13	1/5	0/12	0/31	2/30	0/8	0/6	0/8	0/7	0/1	4/121
**2017**		-	-	0/1	0/21	0/26	0/18	0/3	0/13	0/5	0/10	0/4	0/6	0/107
**2018**		-	0/2	0/2	0/13	0/36	0/20	0/2	0/9	0/10	0/4	-	0/9	0/107
**2019**		-	1/8	0/19	1/15	0/20	0/19	1/32	3/25	-	-	-	-	6/138

## Supplementary Materials

The following are available online at https://www.mdpi.com/2076-2607/8/6/844/s1, Table S1: Bat samples information, Table S2: Information of the positive sample.
