# Supplementary material for: The Epidemiological Characteristics of the Korean Bat Paramyxovirus between 2016 and 2019
Source: Microorganisms. 2020 Jun 4;8(6):844. doi: 10.3390/microorganisms8060844 (PMC7356101; doi:10.3390/microorganisms8060844)
Supplement: Supplementary file 1 [file microorganisms-08-00844-s001.pdf]

The epidemiological characteristics of the Korean bat paramyxovirus between 2016 and 2019

Seong Sik Jang[1], Ji Yeong Noh[1], Van Thi Lo[2,3,] Yong Gun Choi[4], Sun-Woo Yoon[2,3], Dae Gwin Jeong[2,3], Hye Kwon Kim[1*]

[1] Department of Microbiology, College of Natural Sciences, Chungbuk National University, Cheongju, Republic of Korea

[2] Infectious Disease Research Center, Korea Research Institute of Bioscience and Biotechnology, Daejeon, Korea

[3] Bio-Analytical Science Division, University of Science and Technology (UST), Daejeon, Republic of Korea

[4] The Korean Institute of Biospeleology, Daejeon, Republic of Korea

*Correspondence: khk1329@chungbuk.ac.kr; Tel.: +82-43-261-2302

Supplementary table S1. Bat samples information

Cases	Province	Region	Site name	Collected date	No. of fecal samples
1	Chungbuk	Danyang	G cave	10-Apr-16	2
				16-Apr-16	2
				27-Jun-16	11
				13-Oct-16	3
2	Chungbuk	Danyang	HSA bridge	27-Jun-16	4
				18-Jul-16	2
				11-Sep-16	2
				02-Oct-16	1
				10-Jun-17	2
				20-Jul-17	3
				25-Apr-19	3
3	Chungbuk	Danyang	HA bridge	28-Jun-16	3
4	Chungbuk	Danyang	SM cave	28-Jun-16	1
5	Chungbuk	Chungju	CS cave	28-Jun-16	5
				20-Apr-17	3
				18-Jul-19	10
6	Chungbuk	Goesan	LK cave	18-Jul-19	6
7	Chungnam	Boryeong	BR abandoned mine	29-May-17	3
8	Chungnam	Geumsan	SK abandoned mine	20-Mar-19	11
9	Gangwon	Jungsun	HPDPN cave	20-Mar-16	1
10	Gangwon	Jungsun	SM cave	19-Jun-16	3
11	Gangwon	Jungsun	WP cave	24-Sep-16	1

12	Gangwon	Jungsun	BR cave	25-Sep-16	1
				22-Aug-19	2
13	Gangwon	Jungsun	GAU cave	16-Oct-16	1
14	Gangwon	Jungsun	YM cave	23-Oct-16	1
				20-Nov-16	3
15	Gangwon	Donhae	SH temple	13-Jul-16	2
16	Gangwon	Yeongwol	GS cave	16-Jul-16	2
				24-Sep-16	2
17	Gangwon	Yeongwol	WS bridge	19-Jul-16	5
18	Gangwon	Yeongwol	OJ cave	19-Jul-16	12
19	Gangwon	Pyeongchang	BR cave	16-Jul-16	2
20	Gangwon	Pyeongchang	S cave	20-Jul-16	5
				11-Apr-19	2
				09-May-19	2
				04-Jun-19	2
				01-Jul-19	4
				21-Aug-19	3
21	Gangwon	Pyeongchang	GCS cave	20-Jun-17	5
				04-Aug-17	3
				17-Oct-17	2
				10-Apr-19	4
				09-May-19	3
				03-Jun-19	3
				02-Jul-19	3
				21-Aug-19	3

22	Gangwon	Pyeongchang	MS cave	22-Aug-17	1
23	Gangwon	Pyeongchang	JA cave	21-Apr-18	3
				10-Aug-18	7
				17-Feb-19	3
				23-Mar-19	2
				12-Apr-19	3
				10-May-19	6
				04-Jun-19	2
				02-Jul-19	2
				20-Aug-19	4
				24-Aug-19	3
24	Gangwon	Pyeongchang	BL cave)	11-Apr-19	3
				10-May-19	2
				01-Jul-19	1
				21-Aug-19	1
25	Gangwon	Samcheok	JBS cave	11-Jun-17	3
26	Gangwon	Samcheok	CBJ cave	24-Sep-17	4
27	Gangwon	Samcheok	GH cave	28-Oct-17	4
				26-Nov-17	4
28	Gangwon	Samcheok	SDSJ cave	29-Oct-17	1
29	Gangwon	Samcheok	GJLSJ cave	20-May-18	1
30	Gangwon	Samcheok	S cave	17-Jun-18	2
31	Gangwon	Samcheok	HWS cave	27-Jun-18	3
32	Gangwon	Samcheok	SH cave	28-Jun-18	4
33	Gangwon	Samcheok	PM temple	23-Jul-18	1

34	Gangwon	Samcheok	MG cave	30-Sep-18	4
35	Gangwon	Samcheok	HS cave	20-Oct-18	1
36	Gangwon	Samcheok	SG cave	19-May-19	2
37	Gangwon	Samcheok	SGJ cave	16-Jun-19	4
38	Gangwon	Samcheok	CGLB cave	18-Aug-19	1
39	Gangwon	Samcheok	YHH cave	19-Aug-19	2
40	Gyeongbuk	Munkyung	HG abandoned mine	16-Mar-16	3
41	Gyeongbuk	Munkyung	BGA cave	18-May-16	1
				21-Apr-17	6
42	Gyeongbuk	Munkyung	BGS cave	18-May-16	4
				21-Apr-17	4
				23-Dec-17	1
				25-Dec-18	4
				23-Mar-19	5
				20-May-19	2
				13-Jul-19	3
43	Gyeongbuk	Munkyung	UL cave	29-May-16	2
				24-Mar-19	1
44	Gyeongbuk	Munkyung	BMA cave	19-Mar-17	1
45	Gyeongbuk	Munkyung	GDA cave	23-Dec-17	1
46	Gyeongbuk	Munkyung	MS cave	21-May-19	3
				13-Jul-19	3
47	Gyeongbuk	Youngju	deserted house	10-May-16	1
48	Gyeongbuk	Kyungju	SC bridge	15-Jun-16	3
49	Gyeongbuk	Bonghwa	NBI cave	23-Apr-17	2

50	Gyeongbuk	Bonghwa	DLN cave	22-Apr-18	3
				25-May-18	6
51	Gyeongbuk	Uljin	SL cave	22-Jul-18	1
52	Gyeonggi	Anseong	CL abandoned mine	20-Apr-17	3
				23-May-18	5
53	Gyeonggi	Anseong	SPL abandoned mine	20-Apr-17	2
				23-May-18	6
54	Gyeongnam	Hapchoen	BT cave	16-Mar-16	2
				18-May-16	2
				28-Aug-16	3
				08-Oct-16	2
55	Incheon		HNJSJ cave	24-May-18	15
				26-Aug-19	3
56	Jeju Island	Jeju	USJ cave	21-Mar-16	1
				27-Jun-19	5
57	Jeju Island	Jeju	MJ cave	21-Mar-16	1
				25-Jun-19	1
58	Jeju Island	Jeju	artificial cave	23-Mar-16	1
59	Jeju Island	Jeju	MSB cave	28-Apr-16	1
60	Jeju Island	Jeju	GR cave	09-Jun-16	1
				24-Aug-17	5
61	Jeju Island	Jeju	WH cave	19-Aug-16	1
				05-Nov-16	3
62	Jeju Island	Jeju	KNBLM cave	20-Aug-16	2
				22-Aug-17	4

63	Jeju Island	Jeju	SCS cave	29-Jun-17	1
64	Jeju Island	Jeju	GUDD cave	09-Sep-17	1
65	Jeju Island	Jeju	GNAS cave	14-Dec-17	4
66	Jeju Island	Jeju	MSM cave	06-Apr-18	3
67	Jeju Island	Jeju	CJD cave	31-May-18	3
68	Jeju Island	Jeju	SSB cave	28-Aug-18	1
69	Jeju Island	Jeju	CSS cave	06-Sep-18	2
70	Jeju Island	Jeju	GNS cave	26-Jun-19	2
71	Jeju Island	Seogwipo	artificial cave	22-Mar-16	1
72	Jeju Island	Seogwipo	HDR cave	23-Mar-16	1
				14-Dec-16	1
				01-Apr-18	4
				24-Oct-18	3
				13-Feb-19	2
73	Jeju Island	Seogwipo	SAOL cave	18-Aug-16	2
				09-Apr-17	1
				29-Jun-17	7
				12-Oct-17	1
				13-Oct-17	1
				14-Oct-17	1
				13-Jun-18	1
				20-Dec-18	5
				11-Feb-19	3
				12-Aug-19	3
74	Jeju Island	Seogwipo	SS cave	05-Nov-16	1

75	Jeju Island	Seogwipo	BLL cave	22-Mar-18<br>13-Jun-18	2<br>4
76	Jeju Island	Seogwipo	PSNJ cave	28-Aug-18	1
77	Jeonnam	Jindo	SJ cave	25-Mar-16<br>24-May-17	2<br>10
78	Jeonnam	Hampyeong	JC cave	25-May-16<br>23-May-17	2<br>8
79	Jeonnam	Hampyeong	GSAL cave	23-May-17	5
80	Jeonnam	Hampyeong	JCJ cave	22-Jun-18	1
81	Jeonnam	Muan	IS cave	13-Feb-18	2
82	Jeonnam	Muan	SP abandoned mine	21-Jun-18	3
83	Jeonnam	Sinan	SOD abandoned mine	21-Jun-18	2
84	Jeonnam	Sinan	DC island	11-Sep-18	2
85	Jeonnam	Sinan	UI island	11-Sep-18	2
			Total		473

The abbreviations list: G: Geum, HSA: Ha Seon Am, HA: Heung Am, SM: Seol Ma, CS: Cheong Seong, LK: Lee Kang , BR: Bo Ryeong, SK: Seong Kog, HPDPN: Hwa Pyo Dong Pi Nan, SM: Saem Mul, WP: Wol Pyeong, BR: Bi Ryong, GAU: Gul A U , YM: Ya Mi , SH: Sam Haw ,GS: Go Ssi, WS: Wa Seong, OJ: Ong Jeong, BR: Baeg Ryong, S: Ssang, GCS: Gwang Cheon Seon, MS: Myo San, JA: Jang Am, BL: Ba Lam, JBS: Jo Bi Seon, CBJ: Chil Bat Jae, GH: Go Hyeol, SDSJ: Such Dun Su Jig, GJLSJ: Go Ja Li Su Jig, S: Seong, HWS: Ha Wol San, SH: So Han, PM: Pye Mag, MG: Mai Gol, HS: Hwan Seon, SG: Sa Gog, SGJ: Seog Gae Jae, CGLB: Cha Gu Li Bum, YHH: Yeon Ha Ha, HG: Ho Gye, BGA: Bu Gog Am, BGS: Bu Gog Sus, UL: U Lo, BMA: Bing Mo Am, GDA: Ga Do Am, MS: Mo San, SC: Sin Cheon, NBI: Neo Beng I, DLN: Deu Leu Ne, SL: Seong Lyu, CL: Cheong Lyong, SPL: Sin Pyeong Li, BT: Be Ti, HNJSJ: Ha Neul Jae Su Jeong, USJ: Us San Jeon, MJ: Man Jang, MSB: Myo San Bong, GR: Gu Rin, WH: Wa Heul, KNBLM: Kim Nyeong Bil Le Mos, SCS: Sin Chang Seong, GUDD: Ge Us Du Dug, GNAS: Go Neng A Sul, MSM: Mog Su Mul, CJD: Chu Ja Do, SSB: Seo Seon Bong, CSS: Chu Seong San, GNS: Gim Nyeom Sa, HDR: Han Deu Reu, SAOL: Seos Al O Leum, SS: Seong San, BLL: Beol La Li, PSNJ: Pyo Seon Nab Jag, SJ: Sib Ja, JC: Jeong Chang, GSAL: Geum Seong A Lae, JCJ: Jeong Chang Jin, IS: I Seong, SP: Seok Po, SOD: Sa Og Do, DC: Do Cho, UI: U I

Supplementary table S2. Information of the positive sample.

Sample name	Accession number	Collected date	Region	Site name	Type of sample	Identification of bat species
B16-6	MT264743	16 March 2016	Hapcheon	BT cave	Feces	Not determined
B16-40	MT264744	16 April 2016	Danyang	G cave	Feces	Not determined
B16-148	MT264745	19 July 2016	Yeongwol	OJ cave	Feces	*Myotis macrodactylus*
B16-154	MT264746	20 July 2016	Pyeongchang	S cave	Feces	Not determined
B19-3	MT264748	11 February 2019	Seogwipo	SAOL cave	Feces	Not determined
B19-33	MT264749<br>MT212726	10 April 2019	Pyeongchang	GCS cave	Feces	Not determined
B19-112	MT264750	18 July 2019	Goesan	LK cave	Feces	*Myotis petax*
B19-145	MT264751	20 August 2019	Pyeongchang	JA cave	Feces	*Miniopterus schreibersii*
B19-151	MT264752<br>MT230548	21 August 2019	Pyeongchang	S cave	Feces	*Myotis macrodactylus*
B19-152	MT264753<br>MT277365	21 August 2019	Pyeongchang	S cave	Feces	*Myotis macrodactylus*
